# Assessing the stress-relief impact of an art-based intervention inspired by the broaden-and-build theory in college students

**DOI:** 10.3389/fpsyg.2024.1324415

**Published:** 2024-01-31

**Authors:** Chen Liu, Yuan Xie, Yiwen Xu, Zhenhai Song, Jiayi Tang, Junjie Shen, Zhou Jiang, Chao Shen, Xingya Zhan, Chu Zheng

**Affiliations:** ^1^School of International Education, Xuzhou Medical University, Xuzhou, China; ^2^School of Public Health, Xuzhou Medical University, Xuzhou, China; ^3^School of Basic Education and Art, Shandong Vocational College of Industry, Zibo, China; ^4^Department of Biostatistics, School of Public Health, Xuzhou Medical University, Xuzhou, China; ^5^Department of Immunization Program, Nanjing Municipal Center for Disease Control and Prevention, Nanjing, China; ^6^School of Public Health, Nanjing Medical University, Nanjing, China

**Keywords:** PSS, college students, stress relief, art-making, tension

## Abstract

**Background and objectives:**

This study’s primary objective is to investigate the impact of art-making on the mental well-being of college students, who often experience heightened stress during their initial university years.

**Methods:**

Employing a comprehensive methodology, combining interviews and the Perceived Stress Scale (PSS), the research aimed to assess whether a four-week art-making intervention can effectively alleviate stress levels among college students. In the experimental group, participants engaged in a variety of art-making activities, including freehand drawing, clay modeling, and crafting.

**Results:**

The results revealed that, in the pre-test, there were no significant differences between the experimental and control groups for each assessed indicator. However, in the post-test, significant differences emerged across all indicators. Further analysis demonstrated a significant reduction in stress perception among the experimental group participants between the pre-test and post-test phases.

**Conclusion:**

In conclusion, this study provides compelling evidence that art-making has the potential to foster positive personal development and significantly reduce stress levels among college students.

## 1 Introduction

Maintaining a healthy mental state is imperative for college students to complete their studies and adapt to society. Medical students, in particular, encounter heightened pressure due to the demanding coursework, which takes a toll on their mental well-being. Besides, freshmen, navigating the transition from high school to university, experience changes in their learning style and living environment, making them vulnerable to psychological challenges (Ning et al., 2022). Thus, it becomes paramount to address the mental health concerns of medical freshmen.

Stress represents a cascade of physiological responses triggered when an individual perceives physical, mental, emotional, or spiritual threats (Seaward, 2008). A comprehensive study investigated the prevailing stress levels among Chinese undergraduates, revealing that nearly all surveyed students experienced psychological stress, with over half of them enduring moderate to severe stress levels (Lin et al., 2006). When seeking strategies to alleviate stress, scholars abroad have advocated for the use of art-making methods, particularly the psychotherapeutic approach of mandala painting. Their findings consistently demonstrate the effectiveness of art-making in reducing stress and anxiety. Remarkably, art-making involves a creative process that entails the observation, experience, research, analysis, selection, processing, and refinement of real-life elements through a series of non-verbal creative techniques. It therefore serves as a means to visualize individuals’ internal and subconscious content (Huihui, 2017). Through a space for free exploration and creative work, art therapy can provide the following: promote things that are difficult or impossible to express in words; Evokes many defense mechanisms; Induce responses that may be ambiguous or confusing. Therefore, art therapy and art making can be used as an effective means to address stress for individuals with emotional or psychological difficulties, including those who have difficulty expressing their emotions directly (Timulak et al., 2017). Cho (2022) research shows that paint-centered art therapy can help students improve their self-awareness and expression skills, increase in-group intimacy, reduce community stress, and improve quality of life. Together, these previous studies have shown that group art therapy has a positive effect on reducing stress (Yin and Ko, 2023).

In his study, De Morais actively engaged psychiatric patients with clay, demonstrating its effectiveness in reducing anxiety among them. He argued that art therapy, serving as a non-verbal means of expressing emotions, plays a pivotal role in anxiety reduction (de Morais et al., 2014). Creative art therapy is a concentrated form of psychotherapy that includes a wide range of activities (such as meditation, body movement, art, dance, acting, painting, expression, puppetry, and storytelling) that can reduce depression, anxiety, and stress in patients (Eum and Yim, 2015; Kulari, 2017). In addition, it is a novel therapy that uses a creative process to help individuals explore their feelings and emotions, providing new ways to gain personal insight and develop coping skills (Kulari, 2017). Similarly, David A. Sandmire employed mandala coloring, clay work, and drawing as interventions for students in his study, affirming that art-making enhances vagal activity, bolsters concentration during the creative process, and diminishes anxiety levels (Sandmire et al., 2016). Numerous investigations have highlighted the utility of art-making in treating various illnesses, effectively alleviating symptoms of anxiety and concentration deficits linked to these conditions (Nainis et al., 2006; Sandmire et al., 2016). In an article exploring the foundations of visual journaling as a means to mitigate stress among medical students, researchers concluded that drawing aids students in visualizing their stressors more effectively, facilitating their transformation into positive emotions (Mercer et al., 2010).

Retrospectively, Coloring Therapy, initially introduced by Belchamber as a crucial aspect of art production (Belchamber, 1997), combines art therapy and meditation techniques, allowing individuals to color intricate geometric patterns to momentarily silence their “inner dialogue,” offering respite from everyday cognitive demands and negative thoughts. Curry and Kasser (2005) noted that although coloring therapy may not encompass all elements of traditional art therapy or meditation, it fosters deep engagement through artistic expression, contributing to anxiety reduction. Additionally, Berberian, a clinical assistant professor in New York University’s (NYU’s) graduate art therapy program, suggested that akin to meditation, coloring enables individuals to disconnect from distracting thoughts, focusing solely on the present moment, thereby alleviating anxiety (Fitzpatrick, 2017). Simultaneously, the American Art Therapy Association distinguishes coloring as separate from art therapy but recognizes it as an active process for externalizing focus, diverting individuals from an unhealthy “inner dialog (Carolan and Betts, 2015). Andrea C found in art therapy-based studies that participants were able to learn specific ways to relax during art therapy, noticed that their physical and mental connections were strengthened, and they were better able to manage stress (Cheshure and Van Lith, 2022). According to Kurt’s research (Fehrman and Fehrman, 2009), therapy involving color delves deeper, suggesting that each color wavelength targets specific areas of the body, eliciting distinct physiological responses that aid in anxiety relief. Chinese scholars conducted a reintervention study involving coloring activities, affirming coloring as a positive psychological intervention (Wang, 2022).

Currently, suggestions for alleviating the stress faced by Chinese college students primarily revolve around music therapy and physical exercise. In contrast, literature on art-based interventions is limited, with most references centering on mandala painting. Building upon these insights, we implemented an art-making program encompassing painting, claywork, handicrafts, and more as an intervention to address college students’ stress levels. Our study aimed to investigate the potential stress-reduction benefits of art-making. In doing so, we embraced the “broaden-and-build theory of positive emotions.”

The “broaden-and-build theory of positive emotions” stands as one of the most influential theory concepts within the realm of positive emotion theory. Originally formulated by Fredrickson this theory emerged from a comprehensive synthesis of prior research and has garnered substantial empirical support, yielding fruitful outcomes. At its core, the theory highlights two primary functions of positive emotions: expansion and construction. Both theoretical frameworks and empirical investigations have demonstrated that positive emotions counteract the physiological consequences of negative emotions. They widen an individual’s scope of attention, cognition, and behavior, fostering more efficient information acquisition and analysis. Furthermore, positive emotions motivate ongoing knowledge acquisition and the accumulation of experiences conducive to goal attainment, while also sparking novel problem-solving strategies and creative initiatives. This embodies the “expansion” function of positive emotions. Building upon this expansion function, positive emotions play a vital role in constructing and bolstering individuals’ enduring resources, encompassing physical, intellectual, psychological, and social aspects, ultimately enhancing their subjective well-being (Xiaowei, 2013).

This study actively fostered positive emotions among the intervention subjects, expanding their cognitive and behavioral capabilities, encouraging the development of novel problem-solving approaches, and inspiring them to engage in creative activities. Concurrently, positive emotions facilitated the establishment of enduring social resources for the intervention subjects, enabling them to share joy and excitement with others through communication, collaboration, and cooperation, thereby strengthening their social bonds and attachments. Consequently, individuals were able to pursue healthy self-development, contributing to the attainment of personal goals and enhancing individual adaptability. As a result, we utilized art creation as a behavioral technique to examine the impact of art-making on stress perception, guided by the broaden-and-build theory of positive emotions.

## 2 Methods

### 2.1 Participants

In this study, we recruited 90 students from a pool of approximately 1,000 medical school students consisting of 45 males and 45 females who willingly volunteered, with an average age of 18 ± 1 years. These participants were then randomly divided into two groups, consisting of 45 individuals each: an experimental group and a control group. Registration occurred through roll-call, and at the end of the intervention, participants who had completed less than 90% of the tasks or were absent from the control group were excluded. Consequently, the final tally comprised a total of 70 participants, with 33 in the experimental group and 37 in the control group.

### 2.2 Recruitment

We sent an email to eligible freshmen through an on-campus platform, and students who expressed interest were sent a follow-up research abstract and consent form via email. Once they had signed the consent form, participants were put on schedule for a face-to-face orientation session for initial communication. All interviewers and data collectors were professionally trained.

### 2.3 Design and intervention description

**Figure 1:**
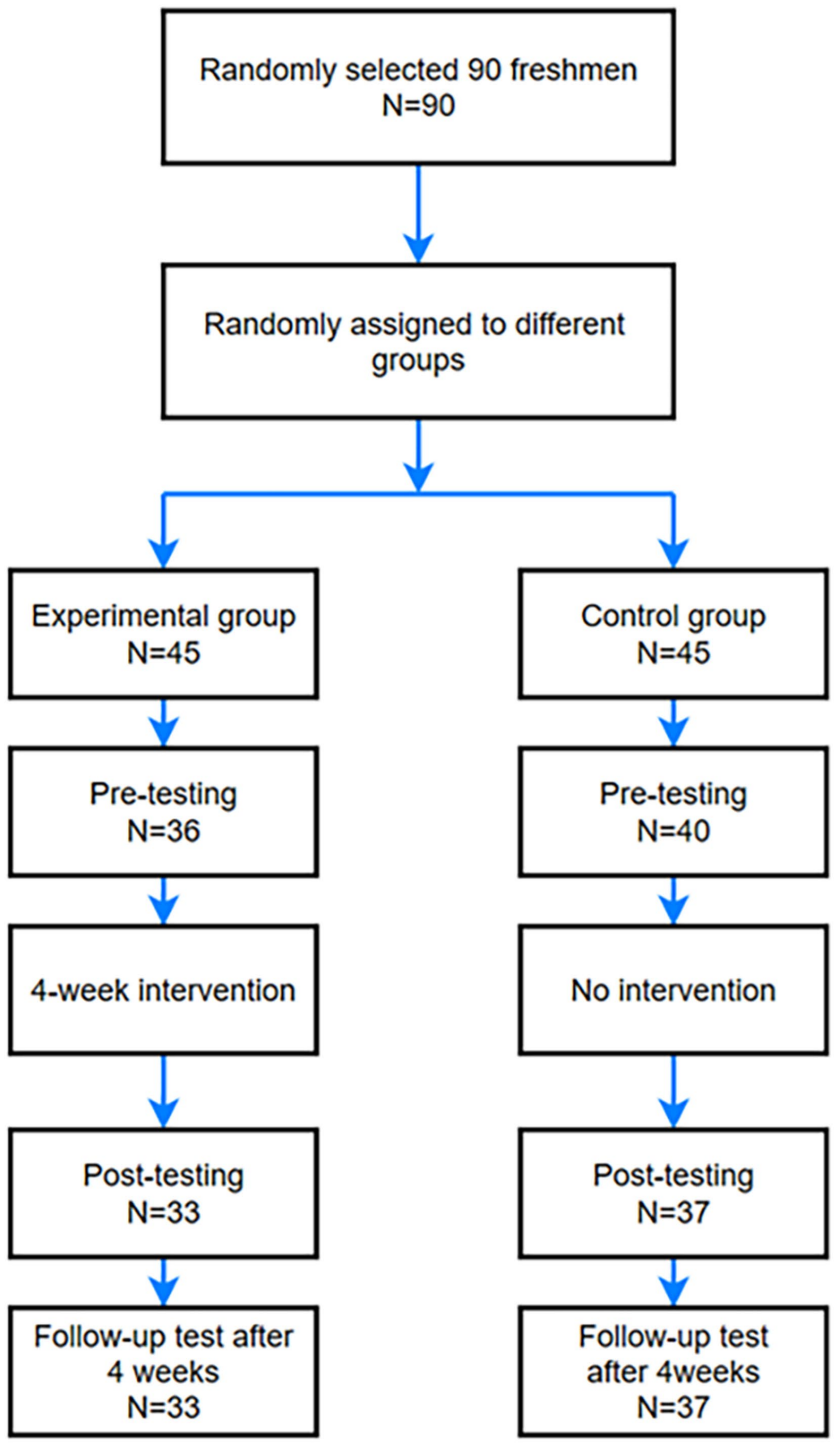
Intervention process.

This study employed a design involving pre-tests, post-tests, and follow-up tests to compare the experimental and control groups. Initially, both the experimental and control groups underwent a pre-test using the Perceived Stress Scale (PSS) (Yang and Huang, 2003) before the commencement of the 4-week intervention trial. Subsequently, the experimental groups received the intervention throughout four sessions spanning four weeks, while the control groups did not receive any intervention during this period. Hence, the intervention process for the experimental groups is illustrated in the following flowchart. After the 4-week intervention trial, a post-test was administered, followed by a follow-up test after week 8 (Figure 1).

The experimental groups participated in an art-making course spanning four weeks, with each session lasting 2.5 h per week. Through interviews with the students, we identified the primary sources of stress among university students, which informed the design of the following handicraft course program. We are divided into the following four stages. In the first session, we had a clay-making activity where we used ultralight clay and kneaded our favorite shapes. In the second session, we had a decorating activity where we decorated small wooden boards with butterscotch glue and finished clay products. In the third session, we had a bookmark-making activity, where we used botanical specimens that were made into small bookmarks through the process of making bookmarks. In the fourth session, we did a canvas bag painting activity, where we used acrylic paints to paint the canvas bag.

Our four intervention sessions emphasize the following: (1) Art-making can enhance the concentration of college students and integrate them into the creative process, thereby reducing anxiety associated with stressors and promoting relaxation. (2) Various art-making techniques enable individuals to express and release their ideas in ways distinct from language, effectively contributing to stress relief.

### 2.4 Data collection, measures

The intervention experiment employed a pre-test, post-test, and follow-up design, comparing control and experimental groups with the dependent variable being the score of the Perceived Stress Scale. Initially, participants underwent a pre-test to assess their stress levels upon recruitment, followed by the completion of informed consent forms. They were then randomly assigned to either the experimental or control conditions, with the control group not engaging in any exercises. A total of 90 questionnaires were distributed during the pre-test, all of which were recovered, and 76 questionnaires were deemed valid (36 for the experimental groups and 40 for the control groups), resulting in a validity rate of 84.4%. Remarkably, the intervention lasted for 28 days, concluding with a post-test using the stress perception scale.

Furthermore, in the post-test phase, 90 questionnaires were distributed. Eventually, we collected 70 questionnaires, all of which were considered valid (33 from the experimental groups and 37 from the control groups), yielding a validity rate of 100%. Notably, during the pre-intervention and post-intervention tests between the groups, there were instances of study participants being absent for various reasons or having too many missing values in the questionnaire tests, leading to some individuals who could not be matched between the pre-test and post-test phases. Ultimately, a total of 70 subjects participated in both the pre-test and post-tests, comprising 33 individuals in the experimental groups and 37 in the control groups. A follow-up test was administered to all 70 participants four weeks later, with 70 questionnaires distributed and returned, all of which were valid, resulting in a 100% validity rate. All 70 individuals took part in all three tests, with 33 in the experimental groups and 37 in the control groups.

We assessed stress levels using the PSS. This widely recognized 14-item scale serves as a primary psychological tool for evaluating stress perception, with sample questions like “In the last month, how often have you been upset because of something that happened unexpectedly?” Afterward, participants rated each item on a 5-point Likert scale (1 = Never to 5 = Very often). Intriguingly, items 4, 5, 6, 7, 9, 10, and 13 were positively phrased and thus reverse scored. Whereas, higher scores on the scale indicated greater perceived stress. The scales demonstrated strong statistical reliability (Cronbach’s alpha = 0.88). We have subdivided the stress perception scale into two subscales: tension and loss of control. Stress arises from a certain stimulus event, and when encountering a stimulus event that the individual believes to be out of his/her control, tension arises, which can lead to a series of physiological changes for the person concerned. The sense of loss of control represents the individual’s own perception and evaluation of the stimulus event: that is, when the individual believes that the stimulus event is out of his or her control, he or she will experience stress. Tension represents an individual’s internal experience, and the two subscales of loss of control (Cronbach’s alpha = 0.899) and tension (Cronbach’s alpha = 0.854) which have good reliability can better represent the perception of stress (Shaolin, 2013).

### 2.5 Statistical analysis

In preliminary analyses and demographic information, continues variables are displayed as *M ± SD,* and categorical variables are displayed as numbers frequency (percentages). Categorical variables in different groups were compared by chi-square test when appropriate. In order to analyze the differences between groups, we used independent samples *t*-tests. Prior to the repeated measures ANOVA, the variance of the difference between the different measurements was first tested for equality using the spherical test, and after passing the test, the experimental treatment (control vs. experimental) was used as the between-group variable, and the time factor (pre-test vs. post-test vs. follow-up) was used as the within-group variable to analyze whether tension, loss of control, and stress perceptions changed over time by repeated-measures analysis of variance (ANOVA). Multiple comparisons of tension and stress perception scores with validated outcomes and Least Significant Difference (LSD) calibration were performed to identify specific periods of change. The critical alpha for internal validity analyses was set at 0.05. All analyses were conducted using SPSS version 26.

## 3 Results

### 3.1 Preliminary analyses and demographic information

The chi-square test results indicated no significant differences in age, gender, or specialization between the control and experimental groups before implementing the intervention (*p* > 0.05). Additionally, an analysis of the pre-intervention questionnaires revealed no substantial disparities in tension, loss of control, and stress perception (*p* > 0.05), underscoring the similarity in stress levels among the sampled students and ensuring the accuracy of the intervention results (Table 1).

**Table 1:** Baseline characteristics.

Variable	Control group (*N* = 37)	Experimental group (*N* = 33)	*χ*^2^/*t*	*p-*value	*df*
Age	18 ± 1	18 ± 1			
Gender			0.002	0.967	1
Male	17 (45.9%)	15 (45.5%)			
Female	20 (54.1%)	18 (54.5%)			
Subjects			0.000	1.000	1
Medical-related	35 (94.6%)	32 (97.0%)			
Medical-unrelated	2 (5.4%)	1 (3.0%)			
Stress perception	6.00 ± 1.06	6.30 ± 0.70	−1.353	0.180	68
Tension	3.16 ± 0.58	3.32 ± 0.51	−1.208	0.231	68
Loss of control	2.84 ± 0.63	2.98 ± 0.41	−1.067	0.290	68

Stress perception, tension, and loss of control as *t*-tests.

### 3.2 Between-group comparison

#### 3.2.1 Comparison of stress indicators between control groups and experimental groups at week 4

The results indicate that following the experiment, students in the experimental group experienced a significant reduction in stress perception compared to the control groups, with a notable between-group difference (*t* = 4.061, *p* < 0.01). Regarding tension, students in the experimental groups exhibited a significant decrease in tension in comparison to the control groups, emphasizing the meaningfulness of the results (*t* = 4.829, *p* < 0.01). However, there were no significant changes observed in terms of loss of control following the intervention (*p* > 0.05) (Table 2).

**Table 2:** Comparison of 4-week stress indicators.

Assessment point	Control group (*N* = 37)		Experimental group (*N* = 33)		*df*	*t*	*p-*value
	*M*	*SD*	*M*	*SD*			
Stress perception	6.12	0.90	5.31	0.76	68	4.061	0.001*
Tension	3.20	0.65	2.55	0.46	68	4.829	0.001*
Loss of control	2.91	0.71	2.76	0.50	68	1.034	0.305

*p < 0.05.

#### 3.2.2 Comparison of stress indicators between control groups and experimental groups at week 8

The results indicated that during the follow-up period, students in the experimental group continued to experience significant reductions in both stress perception (*t* = 2.145, *p* < 0.05) and tension (*t* = 3.307, *p* < 0.01) compared to the control groups, demonstrating notable between-group differences. Conversely, there were no significant changes observed in terms of loss of control following the intervention (*p* > 0.05). It indicates that the experimental effect is persistent (Table 3).

**Table 3:** Comparison of 8-week stress indicators.

Assessment point	Control group (*N* = 37)		Experimental group (*N* = 33)		*df*	*t*	*p*-value
	*M*	*SD*	*M*	*SD*			
Stress perception	6.09	1.15	5.49	1.18	68	2.145	0.035*
Tension	3.19	0.58	2.74	0.55	68	3.307	0.002*
Loss of control	2.90	0.75	2.75	0.72	68	0.837	0.405

*p < 0.05.

### 3.3 Within-group comparison

#### 3.3.1 Comparison of changes in stress indicators in experimental groups

Tension, loss of control and perception satisfaction Mauchly’s Test of Sphericity (*p* > 0.05). The results demonstrated significant changes in stress perception (*df* = 2, *F* = 4.172, *p* = 0.017) (Table 4) and tension (*df* = 2, *F* = 7.615, *p* = 0.001) (Table 5) throughout the experimental and follow-up periods. Conversely, no significant changes were observed in loss of control before, during, and after the experimental and follow-up phases (*df* = 2, *F* = 0.419, *p* = 0.658) (Table 6).

**Table 4:** Repeated measures ANOVA for pressure perception.

Source of variation	*df*	*SS*	*MS*	*F*	*p*
Group factor	1	7.206	7.206	7.203	0.009*
Time factor	2	7.835	3.917	4.172	0.017*
Time ×group	2	12.019	6.009	6.400	0.002*
Individual error	68	68.023	1.000		
Repeated measurement error	136	127.702	0.939		

*p < 0.05.

**Table 5:** Repeated measures ANOVA for tension.

Source of variation	df	SS	MS	*F*	*P*
Group factor	1	5.250	5.250	18.605	<0.001*
Time factor	2	5.059	2.530	7.615	0.001*
Time ×group	2	6.275	3.137	9.444	<0.001*
Individual error	68	19.190	0.282		
Repeated measurement error	136	45.180	0.332		

*p < 0.05.

**Table 6:** Repeated measures ANOVA for loss of control.

Source of variation	*df*	*SS*	*MS*	*F*	*p*
Group factor	1	0.154	0.154	0.365	0.548
Time factor	2	0.328	0.164	0.419	0.658
Time ×group	2	0.959	0.480	1.225	0.297
Individual error	68	28.771	0.423		
Repeated measurement error	136	53.272	0.392		

Tension and stress perception were statistically significant, hence the next step in the study. For multiple comparisons between the stages of the experimental group, we calibrated using the LSD method.

Surprisingly, the results revealed a significant difference between tension levels before and after the intervention, as well as between the pre-intervention and follow-up periods (*p* < 0.001), underscoring the effectiveness of the intervention. Interestingly, there was no significant difference between tension levels after the intervention and during the follow-up periods (*p* > 0.05), indicating the sustained effect of the intervention (Table 7). Similarly, the results of the stress perception indicated the intervention’s effectiveness, with a lasting impact (Table 8).

**Table 7:** Multiple comparisons of tension in different groups.

Groups	0 week	4 week	8 week	*P*_12_	*P*_13_	*P*_23_
Control group	3.16 ± 0.58	3.20 ± 0.65	3.19 ± 0.59	0.748	0.838	0.917
Experimental group	3.32 ± 0.51	2.55 ± 0.46	2.74 ± 0.55	<0.001	<0.001	0.161
*P*	0.231	<0.001	0.002			

P_12_ and P_13_ indicate *p* values for 0 week compared with 4 week and 8 week, and P_23_ indicates *p* values for 4 week compared with 8 week. Amended by LSD.

**Table 8:** Multiple comparisons of stress perception in different groups.

Groups	0 week	4 week	8 week	*P_12_*	*P_13_*	*P_23_*
Control group	6.01 ± 1.06	6.12 ± 0.90	6.09 ± 1.15	0.586	0.740	0.912
Experimental group	6.30 ± 0.70	5.31 ± 0.76	5.49 ± 1.18	<0.001	0.003	0.433
*P*	0.180	<0.001	0.035			

P_12_ and P_13_ indicate *P* values for 0 week compared with 4 week and 8 week, and P_23_ indicates *p* values for 4 week compared with 8 week. Amended by LSD.

#### 3.3.2 Comparison of changes in stress indicators in control groups

The results revealed that before, after, and during the follow-up period, students in the control group displayed no significant changes in any of the factors (*p* > 0.05) (Tables 7, 8).

### 3.4 Effectiveness testing of interventions

#### 3.4.1 Time × group interaction for tension

**Figure 2:**
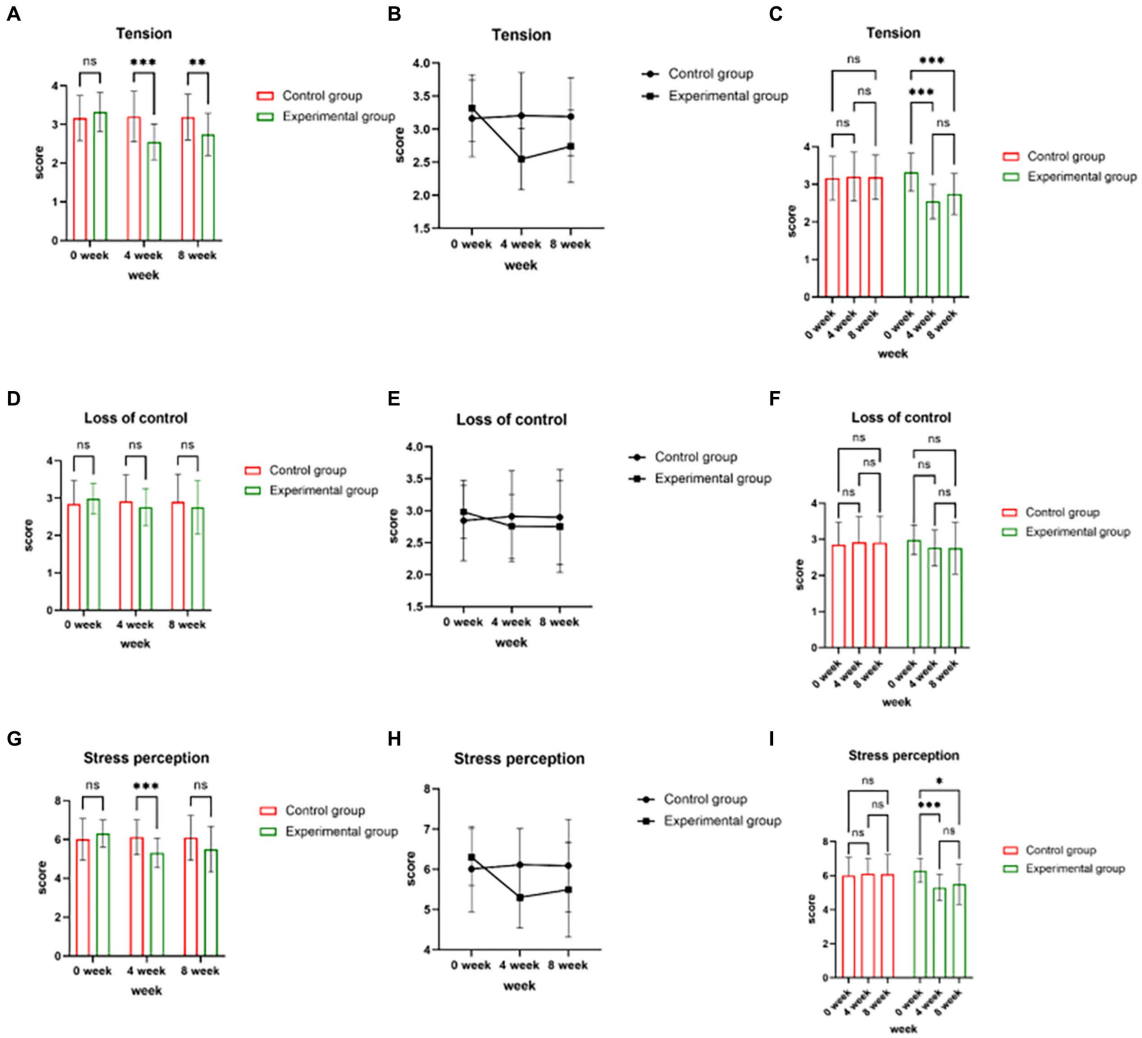
Time and group interaction for stress indicators processes. (**A-C**: stress scores for tension, **D-F**: stress scores for loss of control, **G-I**: stress scores for stress perception).

In repeated measures ANOVA for the experimental and control groups, the distribution of tension satisfaction passed Mauchly’s Test of Sphericity (*p* > 0.05). There was a significant time × group interaction effect for tension (*df* = 2, *F* = 9.444, *p* < 0.001) (Table 5). The difference between the scores of the experimental and control groups on the Tension dimension at weeks 4 and 8 was significant, and the experimental group showed a significant decrease in tension at week 4, which remained at a stable level at week 8 (Figures 2A–C).

#### 3.4.2 Time × group interaction for loss of control

In repeated measures ANOVA for the experimental and control groups, the distribution of loss of control satisfaction passed Mauchly’s Test of Sphericity (*p* > 0.05). There was no significant time × group interaction effect for tension (*df* = 2, *F* = 1.225, *p* = 0.297) (Table 6; Figures 2D–F).

#### 3.4.3 Time × group interaction for stress perception

In repeated measures ANOVA for the experimental and control groups, the distribution of stress perception satisfaction passed Mauchly’s Test of Sphericity (*p* > 0.05). There was a significant time × group interaction effect for stress perception (*df* = 2, *F* = 6.400, *p* = 0.002) (Table 4). The difference between the scores of the experimental group and the control group on the dimension of stress perception was significant at week 4 and the experimental group showed a significant decrease in stress perception at week 4, which remained at a stable level at week 8 (Figures 2G–I).

### 3.5 Subjective assessment results of the art-making courses

In addition to analyzing objective indicators, this study conducted a comprehensive evaluation of the impact of art-making on college students’ stress relief by examining elements related to the characteristics of art-making.

#### 3.5.1 Changes in movement traits and issues

Based on an analysis of the experimental groups’ performance in art-making activities and the interactive sharing sessions, the findings are as follows. Initially, team members tended to replicate the example drawings provided to them. However, as the teams progressed, they began to experiment with new shapes, resulting in a greater diversity of artwork. During the interactive sharing session, team members initially needed passive invitations to the stage, where they simply described their work. Over time, they proactively showcased their creations on stage, sharing their emotional experiences and profound insights gained through their artwork.

#### 3.5.2 Member feedback

The findings derive from subjective feedback collected from members of the experimental group following four art-making sessions. Additionally, the impact of these art-making sessions can be distilled into three key aspects. Firstly, they bolstered members’ capacity for emotional regulation. Interestingly, post-course interviews yielded feedback such as “It has a soothing effect,” “helps in mood relaxation,” “I feel that I can calm down in the process of doing art-making, and alleviate some anxiety,” “In normal life, the fast pace is not very suitable for such art-making at first, but after doing it once or twice, I will gradually learn to enjoy the process. The slow pace of life feels comfortable.”

Secondly, the sessions improved members’ concentration and provided a temporary respite from troubling thoughts. Examples of feedback included, “While engaging in art-making, I will not think about those annoying things, which makes people relax,” and “When art-making, I feel special concentration, usually it is easy to be distracted by electronic products, and I feel relaxed when I concentrate on one thing.” Lastly, the sessions fostered a sense of belonging and mutual support among classmates. Feedback cited instances such as, “I think this course has helped me relieve pressure, and I feel that I have experienced the joy of teamwork in the process of art-making,” “When I shared my ideas, I felt a little embarrassed at first, but later I felt better. And then we did not know each other very well, but through the course, we worked together and strengthened our bond.”

## 4 Discussion

### 4.1 Factors enhancing freshmen’s mental health through art-making

Firstly, art-making constitutes an art program with a role in fostering mental health. On the one hand, within the context of mental health, art education operates as a form of emotional education, mobilizing various mental functions and elevating emotions through exposure to beauty. This process elicits psychological resonate emotionally, and cultivate his temperament through rich inner experience, thus promoting the harmonic development of his own inner self as well as the establishment of a good symbiotic relationship between himself and the external environment. Contemporaneously, art education further contributes to the balanced development of both left and right brain hemispheres, fostering harmony between mind and body, aligning the inner world with the outer world, and unlocking individual potential. Besides, it facilitates the advancement of “harmony between nature and man.”

Given that art education serves as an effective means of expressing personality and oneself, it fosters self-esteem, self-confidence, and personal growth, demonstrating tangible practical value in promoting mental health, and encompassing the essence of mental well-being. Edith Kramer, one of the representative figures in the field of art therapy in the 20th century, established the new concept of art therapy in her practical experience in treating many children. She believes that artistic creation itself has the effect of psychological therapy; With the help of art environment, painting art therapy can help individuals vent psychological problems and bad emotions existing in the subconscious, and the individual psychology will not resist, and this way of catharsis is safe (Yan, 2017). On the other hand, from a psychotherapeutic perspective, art and its educational activities explore, express, and create beauty through mediums such as painting, calligraphy, seal carving, sculpture, and architecture. As individuals engage in these activities, they experience, appreciate, and develop an affinity for beauty. This artistic education nurtures beautiful ideas and, by utilizing the psychological cues of these esthetic concepts, cultivates a vibrant and joyful spiritual realm, infusing life with health and vitality while fostering tranquility of the mind. The suggestive and creative nature of these beautiful ideas effectively mobilizes individuals’ psychological potential, leading to positive emotional reflections in physiological functions. This process generates beneficial stimulation to the cerebral cortex and central nervous system, resulting in enhanced physical and mental well-being (Linyong, 2007). Art works often have the function of washing the soul and edifying sentiment. The same is true of fine arts, which use ingenious ideas, rich colors and proper layout to create works of various styles, where students can get spiritual inspiration and touch, so as to adjust their psychological state and maintain psychological balance. While art has no clear boundaries, teaching through painting can help students better self-cognition (Xiaohong, 2015).

Secondly, students who underwent the art-making intervention exhibited favorable emotional responses to Fredrickson’s broaden-and-build theory, which posits that positive emotions expand an individual’s immediate cognitive and behavioral scope, encompassing heightened attention, cognition, and actions (Fredrickson, 1998). Taken together, both theoretical and empirical studies have demonstrated that positive emotions possess the capacity to counteract the physiological impact of negative emotions, widen the realm of thought and action, enhance problem-solving ability and coping abilities, and cultivate sustainable personal resources, thereby fostering ongoing healthy development. Correspondingly, we observed that individuals with high mental resilience reported experiencing more positive emotions, such as pleasure and interest, before and during high-stress tasks designed to induce negative emotions (Yanmei et al., 2006). This underscores how positive emotions empower individuals to navigate potential adverse situations more effectively, mitigate negative emotional states, enhance psychological resilience, and contribute to mental well-being (Xiaowei, 2013). Students who engaged in the art-making intervention were inclined to adopt a more constructive perspective on things, as their heightened experience of positive emotions broadened their constructive outlook. Consequently, students who participate in diy manual intervention experience more positive emotions, can actively use a variety of shapes, bright colors to create, and can cultivate students’ positive cognitive mode through the artistic image of hand-made products (Yangzhen, 2023).

Thirdly, art-making can enhance the concentration of college students, allowing them to immerse themselves in the creative process, thereby mitigating anxiety stemming from stressors and ultimately reducing pressure, resulting in a sense of relaxation. Building on prior studies, the concept of concentration was propounded by Professor Eugene Gendlin of the University of Chicago in the 1960s as an emotional healing technique aimed at experiencing bodily sensations within specific conditions, which was coined “concentration”(Hinterkopf, 2014). Research has demonstrated that consistent engagement in relaxation and concentration exercises over several weeks can yield various health benefits, including stress reduction, enhanced focus and self-awareness, improved sleep quality, and heightened peace (Danyang and Jiahui, 2022). By setting the theme of handicraft activities, students will gradually complete the handicraft works under the guidance of the lecturer. After each completion of the handicraft work, students will tell the meaning of the work and the production process. This not only enhances the purpose of the students’ learning, but also improves their concentration. With the help of manual activities, students can easily express their inner thoughts and rationally vent their negative emotions in the context of maintaining their concentration (Yangzhen, 2023).

However, the impact of art-making on addressing feelings of loss of control is less pronounced. Intriguingly, loss of control refers to the anxiety stemming from factors beyond one’s control. Equally, implementing time planning strategies for events may be a helpful approach in alleviating feelings of loss of control.

### 4.2 Suggestions on applying art-making to the mental health education of college students

The empirical findings of this study demonstrate that art-making can effectively enhance the mental well-being of college students. In practice, the merits of art-making are evident: it adopts a more engaging and artistic format compared to traditional psychological lectures, making it appealing to students. Furthermore, apart from promoting students’ mental health, it nurtures artistic sensibilities. Contrasted with conventional group counseling, art-making offers a more direct non-verbal avenue for thought and expression. Non-verbal cognition serves to bypass individuals’ subjective consciousness, allowing for a deeper exploration of their inner thoughts (Christenfeld and Creager, 1996). Given the numerous adjustments freshmen undergo during their first semester, which can result in psychological challenges for some students, the implementation of effective mental health education is instrumental in enhancing students’ overall well-being and safety.

However, the integration of art-making courses within colleges and universities also presents certain limitations. During the subject recruitment and interview process, it became evident that not all students possessed the aptitude for art-making in its traditional form, with a general trend of greater receptivity among girls than boys. This discrepancy may stem from the fact that art-making demands a degree of hands-on skill and esthetic sensitivity, areas where girls often exhibit greater proficiency.

### 4.3 Conclusion

In conclusion, this study underscores the significant positive impact of art-making as a novel approach to enhancing the mental health of college freshmen. Through empirical evidence, we have demonstrated that art-making not only effectively promotes emotional regulation, concentration, and positive emotional responses among students but also fosters a sense of belonging and mutual support. Our findings align with the broaden-and-build theory, highlighting the role of positive emotions in expanding cognitive and behavioral capacities. However, this study also has some limitations in the selection of the study population, which came from only one medical college, and we look forward to conducting a more comprehensive study in the future. Meanwhile acknowledging the limitations related to students’ varying aptitudes for art-making, particularly in terms of gender differences, the study underscores the immense potential of art-making as an engaging and creative means of addressing the mental health challenges faced by college freshmen. This research contributes valuable insights into the field of mental health education, advocating for the incorporation of art-making as an innovative and effective tool for improving the psychological well-being of students during their transition to university life.

### 4.4 Recommendations

A recommendation stemming from this study is to incorporate art-making programs into the curriculum of college freshmen as a standard practice for enhancing their mental health. However, it is essential to acknowledge the limitations of this research, particularly the variability in students’ art-making abilities and the gender-related differences in receptivity. To address these limitations, future studies could delve deeper into tailoring art-making interventions to better suit the diverse needs and capabilities of students, thus further enhancing the efficacy of this approach in promoting mental well-being among college freshmen.

## Data availability statement

The original contributions presented in the study are included in the article/supplementary material, further inquiries can be directed to the corresponding author.

## Ethics statement

The study complies with the Declaration of Helsinki and has been approved by the Medical Ethics Committee of Xuzhou Medical University. All students were informed about the purpose of the study and provided written informed consent.

## Author contributions

CL: Data curation, Investigation, Project administration, Supervision, Writing – original draft, Writing – review & editing, Formal analysis, Funding acquisition, Software. YuX: Conceptualization, Data curation, Formal analysis, Investigation, Methodology, Project administration, Software, Validation, Visualization, Writing – original draft, Writing – review & editing. YiX: Conceptualization, Data curation, Formal analysis, Investigation, Methodology, Project administration, Software, Validation, Visualization, Writing – original draft, Writing – review & editing. ZS: Project administration, Supervision, Writing – review & editing. JT: Formal analysis, Software, Writing – review & editing, Project administration. JS: Data curation, Investigation, Writing – original draft, Project administration, Software. ZJ: Project administration, Supervision, Writing – review & editing. CS: Software, Writing – original draft, Data curation, Project administration, Supervision. XZ: Data curation, Methodology, Software, Writing – original draft, Formal analysis, Supervision. CZ: Data curation, Project administration, Software, Supervision, Writing – review & editing.

## References

[ref1] Belchamber B. (1997). Why the mandala? Coloring Therapy. Available at: www.coloringtherapy.com (Accessed January 31, 2003)

[ref2] Carolan R. Betts D.. (2015). The adult coloring book phenomenon. Alexandria, VA: The American Art Therapy Association.

[ref3] Cheshure A. Van Lith T. (2022). A qualitative inquiry comparing mindfulness-based art therapy, mindfulness and neutral clay tasks as a proactive mental health solution for college students. J. Am. Coll Health, 1–11. doi: 10.1080/07448481.2022.2155462 36595633

[ref01] Cho Y. S. (2022). Effect of painting-based group art therapy on acculturative stress, self-expression, and quality of life in international students in Korea. South Korea: Kukje Theological University and Seminary.

[ref4] Christenfeld N. Creager B. (1996). Anxiety, alcohol, aphasia, and ums. J. Pers. Soc. Psychol. 70, 451–460. doi: 10.1037/0022-3514.70.3.451, PMID: 8851740

[ref5] Curry N. A. Kasser T. (2005). Can coloring mandalas reduce anxiety? Art Ther. 22, 81–85. doi: 10.1080/07421656.2005.10129441

[ref6] Danyang L. Jiahui D. (2022). How does the library lnnovate the supply mode of health Information Resources and services: enlightenment from the Stanford health library. J. Acad. Lib. Inform. 40, 131–137. doi: 10.3969/j.issn.1006-1525.2022.02.020

[ref7] de Morais A. Dalécio M. A. N. Vizmann S. Bueno V. L. R. d. C. Roecker S. Salvagioni D. A. J.. (2014). Effect on scores of depression and anxiety in psychiatric patients after clay work in a day hospital. Arts Psychother. 41, 205–210. doi: 10.1016/j.aip.2014.02.002

[ref8] Eum Y. Yim J. (2015). Literature and art therapy in post-stroke psychological disorders. Tohoku J. Exp. Med. 235, 17–23. doi: 10.1620/tjem.235.17, PMID: 25744067

[ref9] Fehrman C. Fehrman K.. (2009). Interior design innovators 1910–1960. Dillsboro, IN: Fehrman Books.

[ref10] Fitzpatrick K. (2017). Why adult coloring books are good for you. CNN Health. Available at: http://edition.cnn.com/2016/01/06/health/adult-coloring-books-popularity-mental-health/

[ref11] Fredrickson B. L. (1998). What good are positive emotions? Rev. Gen. Psychol. 2, 300–319. doi: 10.1037/1089-2680.2.3.300, PMID: 21850154 PMC3156001

[ref12] Hinterkopf E. (2014). Integrating spirituality in counseling: A manual for using the experiential focusing method. London: Jessica Kingsley Publishers.

[ref13] Huihui Y. (2017). Painting art therapy effectiveness research in the students in the Universit entrance exam pressure SIow down. Educ. Teach. Forum 37, 252–254. doi: 10.3969/j.issn.1674-9324.2017.37.111

[ref14] Kulari G. (2017). Art therapy techniques to improve coping strategies in children 7-18 years old with a chronic disease. Universidade NOVA de Lisboa: Portugal.

[ref15] Lin Z. Wen-bo C. Li B. Zhang X.-d. (2006). An epidemiological survey on the psychological stress status for students in13 Chinese colleges. Chin. J. Epidemiol. 27, 387–391. doi: 10.3760/j.issn:0254-6450.2006.05.006 16981331

[ref16] Linyong Z. (2007). The unique function of artistic education in developing the potential of theBrain and promoting mental health. Chin. J. Spec. Educ. 5, 92–94. doi: 10.3969/j.issn.1007-3728.2007.05.017

[ref17] Mercer A. Warson E. Zhao J. (2010). Visual journaling: an intervention to influence stress, anxiety and affect levels in medical students. Arts Psychother. 37, 143–148. doi: 10.1016/j.aip.2009.12.003

[ref18] Nainis N. Paice J. A. Ratner J. Wirth J. H. Lai J. Shott S. (2006). Relieving symptoms in cancer: innovative use of art therapy. J. Pain Symptom Manag. 31, 162–169. doi: 10.1016/j.jpainsymman.2005.07.006, PMID: 16488349

[ref19] Ning Z. Doudou C. Xinyi S. Yiwen C. (2022). A study on the correlation between stress perception and learning burnout of freshmen in medical school. China J. Multimedia Network Teach. 10, 203–206.

[ref20] Sandmire D. A. Rankin N. E. Gorham S. R. Eggleston D. T. French C. A. Lodge E. E.. (2016). Psychological and autonomic effects of art making in college-aged students. Anxiety Stress Coping 29, 561–569. doi: 10.1080/10615806.2015.1076798, PMID: 26222010

[ref21] Seaward B. L. (2008). Stress Management Strategies. Beijing: China Light Industry Press.

[ref22] Shaolin Z. (2013). Review of studies on perceived stress. Business 16, 270–271.

[ref23] Timulak L. McElvaney J. Keogh D. Martin E. Clare P. Chepukova E.. (2017). Emotion-focused therapy for generalized anxiety disorder: an exploratory study. Psychotherapy 54, 361–366. doi: 10.1037/pst0000128, PMID: 29251955

[ref24] Wang S. (2022). Study on the relief effect of coloring activities on anxiety in college students. J. Changchun Normal Univ. 41, 195–200. doi: 10.3969/j.issn.1008-178X.2022.08.036

[ref25] Xiaohong W. (2015). The impact of art education on the physical and mental health of adolescents. Song Yellow River 22:123.

[ref26] Xiaowei Z. (2013). The implications of the "broaden and build" theory of positive emotions for mental health education. J. Chifeng Univ. 29, 132–133. doi: 10.3969/j.issn.1673-260X.2013.14.055

[ref27] Yan L. (2017). The application of art therapy in art education. West Leather 39, 185–186. doi: 10.3969/j.issn.1671-1602.2017.08.156

[ref28] Yang T. Z. Huang H. T. (2003). An epidemiological study on stress among urban residents in social transition period. Zhonghua liu xing bing xue za zhi 24, 760–764. doi: 10.3760/j.issn:0254-6450.2003.09.004 PMID: 14521764

[ref29] Yangzhen W. (2023). Intervention study on the psychological resilience of left-behind children through handicraft activities. Policy Scientific Consult 7, 187–190.

[ref30] Yanmei W. Hailong W. Yinghong L. (2006). The nature and function of positive emotions. J. Capital Normal Univ., 119–122. doi: 10.3969/j.issn.1004-9142.2006.01.023

[ref31] Yin Y. Ko K. S. (2023). The effect of group art therapy on acculturative and academic stress of Chinese graduate students in South Korea. Front. Psychol. 14:1179778. doi: 10.3389/fpsyg.2023.1179778, PMID: 37546469 PMC10397510

